# Loss of Nexmif results in the expression of phenotypic variability and loss of genomic integrity

**DOI:** 10.1038/s41598-022-17845-1

**Published:** 2022-08-15

**Authors:** Caroline Stekelenburg, Jean-Louis Blouin, Federico Santoni, Norann Zaghloul, Elisabeth A. O’Hare, Rodolphe Dusaulcy, Pierre Maechler, Valerie M. Schwitzgebel

**Affiliations:** 1 Pediatric Endocrine and Diabetes Unit, Division of Development and Growth, Department of Pediatrics, Gynecology and Obstetrics, University Hospitals of Geneva, Children’s University Hospital, 6, Rue Willy Donze, 1205 Geneva, Switzerland; 2 Faculty Diabetes Center, Faculty of Medicine, University of Geneva, Geneva, Switzerland; 3 Department of Genetic Medicine and Laboratory, University Hospitals of Geneva, 1211 Geneva, Switzerland; 4 Department of Genetic Medicine and Development, Faculty of Medicine, University of Geneva, 1211 Geneva, Switzerland; 5 Division of Endocrinology, Diabetes and Nutrition, Department of Medicine, University of Maryland School of Medicine, Baltimore, USA; 6 Department of Cell Physiology and Metabolism, University of Geneva Medical Center, 1206 Geneva, Switzerland

**Keywords:** Genetics, Diseases, Endocrinology, Molecular medicine

## Abstract

We identified two *NEXMIF* variants in two unrelated individuals with non-autoimmune diabetes and autistic traits, and investigated the expression of *Nexmif* in mouse and human pancreas and its function in pancreatic beta cells in vitro and in vivo. In insulin-secreting INS-1E cells, *Nexmif* expression increased strongly in response to oxidative stress. CRISPR Cas9-generated *Nexmif* knockout mice exhibited a reduced number of proliferating beta cells in pancreatic islets. RNA sequencing of pancreatic islets showed that the downregulated genes in *Nexmif* mutant islets are involved in stress response and the deposition of epigenetic marks. They include *H3f3b*, encoding histone H3.3, which is associated with the regulation of beta-cell proliferation and maintains genomic integrity by silencing transposable elements, particularly LINE1 elements. LINE1 activity has been associated with autism and neurodevelopmental disorders in which patients share characteristics with *NEXMIF* patients, and can cause genomic instability and genetic variation through retrotransposition. *Nexmif* knockout mice exhibited various other phenotypes. Mortality and phenotypic abnormalities increased in each generation in both *Nexmif* mutant and non-mutant littermates. In *Nexmif* mutant mice, LINE1 element expression was upregulated in the pancreas, brain, and testis, possibly inducing genomic instability in *Nexmif* mutant mice and causing phenotypic variability in their progeny.

## Introduction

The human neurite extension and migration factor (*NEXMIF*) is located on the X-chromosome at locus Xq13.3 in a genomic region of 192 kb that is associated with intellectual disabilities as well as other phenotypes in humans^1^. The X chromosome is highly enriched with genes that are expressed in the brain, and these include *NEXMIF*^2,3^. *NEXMIF* orthologs are highly conserved in mammals, and even in chicken and zebrafish a substantial similarity is observed (61% and 32% sequence identity, respectively). However, at the protein level most of the similarity between human and zebrafish *Nexmif* is absent, with the exception of 112 amino acids in a region of about 250 amino acids (amino acids 310–598)^4^. This 112-amino acid sequence is conserved in all species expressing *Nexmif* and is similar to a small portion of the amino acid sequence of the polymerase zeta subunit *REV3L*^2,5^. DNA polymerase zeta is a low-fidelity DNA polymerase that has an important role in maintaining genomic stability and controlling mutagenesis^6,7^. Further, it has been shown that *Nexmif* is highly expressed in the neurons of the developing mouse brain, where it contributes to the regulation of neurite outgrowth^3,8^. Neurons and beta cells share similar signal transduction pathways and several transcription factors. Several of these transcription factors have been found to be mutated in patients with both mental retardation or brain anomalies and diabetes^9–13^. Based on its expression pattern in the mouse brain and association with mental retardation, NEXMIF has been suggested to have a function similar to that of ATRX, which is involved in chromatin modeling and the maintenance of genomic stability by the silencing of repetitive elements^14^. This, together with the amino acid sequence similarity to REV3L, suggests that NEXMIF might play a role in maintaining genomic stability*.* Here we performed in vitro experiments using INS-1E beta cells and in vivo investigations on a *Nexmif* knockout mouse model to determine the role of Nexmif.

## Results

### Identification of two subjects with a neurodevelopmental syndrome, a *NEXMIF* variant, and diabetes

#### Case 1

The patient, a 15-year-old boy, was born to nonconsanguineous parents of Portuguese origin, at 37 weeks of gestation with a birth weight of 3190 g (P50-P75) and a length of 50 cm (P50). The mother developed gestational diabetes during pregnancy and was treated with insulin. At the age of 4 months, hypotonia was observed, and at 6 months retarded motor development was noticed. At the age of 13 months a first epileptic seizure was seen and treatment with valproic acid was started and pursued until the age of 2 years. Cerebral MRI was within normal limits. A genetic analysis done for severe intellectual disability (IQ 20–34) indicated a 46, XY karyotype, and an array comparative genomic hybridization (aCGH) revealed no duplication or deletion. We identified the pathogenic hemizygous *NEXMIF* variant c.652C>T, p.Arg218* (NM_001008537.3). At the age of 5 years, diabetes was diagnosed after 3 weeks of polyuria and polydipsia with a postprandial glycemia of 26 mmol/L, a capillary beta-hydroxybutyrate level of 0.7 mmol/L (normal < 0.6 mmol/L) and an HbA1c of 91 mmol/mol (10.5%). Autoimmune autoantibodies such as GADA, IAA, IA2A, ZnT8A, and ICA were all negative. The insulin dose was increased gradually from 0.2 to 0.9 U/kg/day. At the age of 15 years the patient is still nonverbal, with autistic behavior.

#### Case 2

The second subject, a 30-year-old women, harbors the pathogenic heterozygous *NEXMIF* c.3470C>A, p.Ser1157* variant associated with mild learning disability, language impairment, epilepsy, and autistic features^15^. She developed autoimmune antibody-negative (GAD and IA2) diabetes at the age of 28 years. Her glucose level at diagnosis was 18.4 mmol/L, with a concomitant capillary beta-hydroxybutyrate level of 3.7 mmol/L. She was first treated with insulin, which could gradually be replaced by an oral antidiabetic treatment with metformin (2 × 1000 mg/day) and gliclazide modified release (2 × 60 mg/day). Her BMI was 34 kg/m^2^ at that time. Her 9-year-old son carries the same *NEXMIF* variant, with severe mental retardation, absence of language, and autistic behavior. The boy has no dysglycemia to date.

### Nexmif is expressed in beta cells

**Figure 1:**
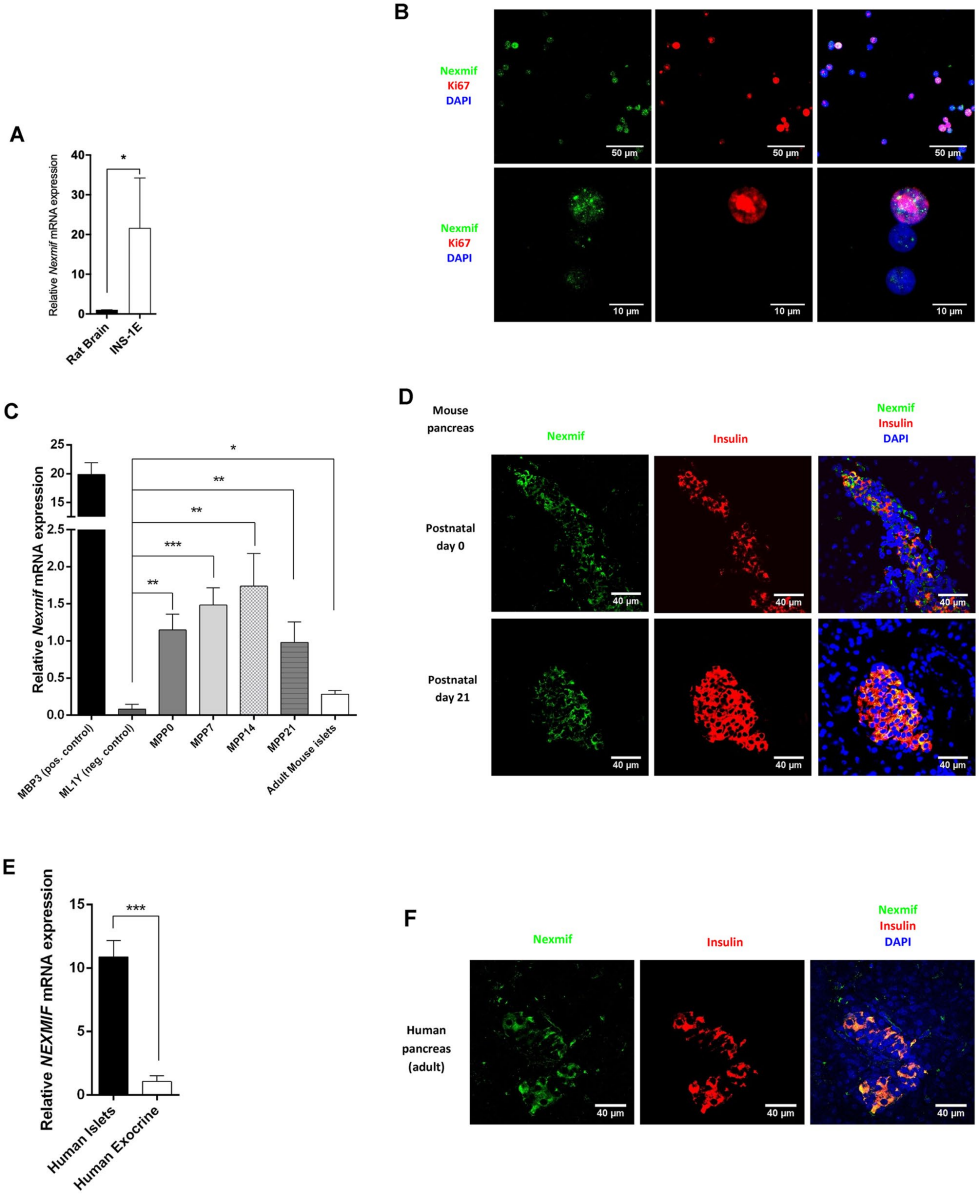
*Nexmif* expression in beta cells in different species and in INS-1E cells. (**A**) *Nexmif* mRNA expression in rat brain (n = 3) and in INS-1E cells (n = 3). (**B**) Nexmif localization (green) in proliferating INS-1E cells marked by Ki67 staining (red). DAPI staining in blue. (**C**) *Nexmif* mRNA expression from left to right in mouse brain at postnatal day 3 (MBP3) (positive control, n = 3); mouse liver at 1 year (negative control, n = 3); mouse pancreas at postnatal day 0 (n = 3), postnatal day 7 (n = 3), postnatal day 14 (n = 3), and postnatal day 21 (n = 3); and adult mouse islets (n = 3). Error bars, standard deviation; n, sample size. (**D**) *Nexmif* localization (green) in mouse pancreas at postnatal days 0 and 21, partially co-staining (yellow) with insulin (red). (**E**) *Nexmif* mRNA expression in human pancreatic endocrine (n = 3) and exocrine tissue (n = 3). (**F**) *Nexmif* localization (green) in human pancreas partially co-staining (yellow) with insulin (red). Error bars, standard deviation; n, sample size. *P ≤ 0.05, **P ≤ 0.01, and ***P ≤ 0.001.

As *Nexmif* was previously described to be highly expressed in the mouse and human brain^2^ (see GTExportal (https://gtexportal.org/home/gene/KIAA2022, accessed Dec 15, 2019), we used brain tissue as a positive control. *Nexmif* was indeed expressed in the rat brain and at even higher levels in rat insulinoma INS-1E cells (Fig. 1A). Co-staining of Nexmif and Ki67 in the INS-1E cells showed that Nexmif was preferentially present in duplicating INS-1E cells (Fig. 1B). We further detected *Nexmif* expression in whole pancreas at postnatal days 0, 7, 14, and 21 and in adult pancreatic islets (Fig. 1C), Nexmif levels decreased two weeks after birth. Similar postnatal decline has already been described in the brain^3^. We used mouse brain at postnatal day 3 as a positive control, as previous studies have shown a maximal expression at this time point^2^. *Nexmif* has been shown to be expressed in most tissues, although GTEx data (human) shows minimal expression in the liver. Accordingly, adult mouse liver was used as a low-expression control. Expression of *Nexmif* was about 10 times lower in the mouse pancreas than in the brain (Fig. 1C). However, Nexmif protein partially co-stained with insulin in islets, with very few Nexmif-positive cells in the exocrine pancreas (Fig. 1D). In human, expression of *NEXMIF* was about 10 times higher in pancreatic islets than in exocrine tissue (Fig. 1E). As it did in mouse pancreas, NEXMIF co-localized partially with insulin in human pancreas (Fig. 1F).

### Nexmif mutant mice

To study the function of *Nexmif* in vivo, we used CRISPR-Cas9 technology to generate *Nexmif* mutant mouse lines. We obtained mice carrying a 50-nt deletion resulting in an early termination codon. Mutant mice were backcrossed with the C57BL6 strain. Nexmif protein was lost from the brain and pancreas of *Nexmif* mutant mice (Additional File 2: Fig. S1).

### Nexmif plays a role in beta-cell proliferation

**Figure 2:**
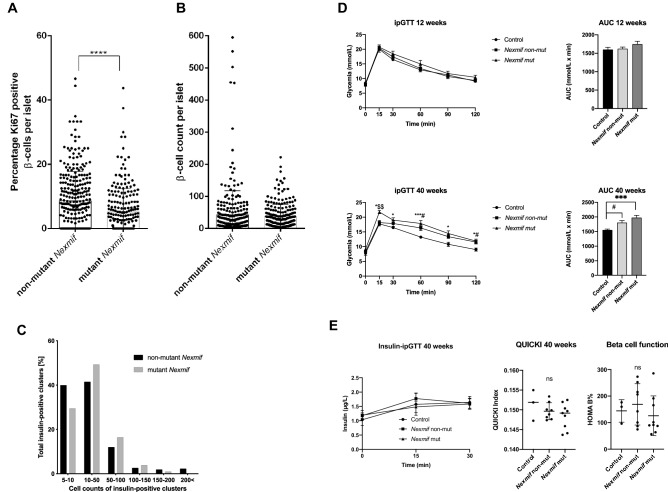
Pancreatic islet proliferation and function in *Nexmif* mutant mice. (**A**) Percentage of proliferating beta cells marked by Ki67 in the pancreatic islets of *Nexmif* non-mutant (n = 3) and mutant mice (n = 3) at postnatal day 21. Error bars indicate the standard deviation. The box plots are presented as median with 25th percentiles (boxes). Two-tailed unpaired Student t-test. ****P ≤ 0.0001. (**B**) Beta-cell counts in pancreatic islets of *Nexmif* non-mutant (n = 3) and mutant mice (n = 3) at postnatal day 21. (**C**) Islet size distribution in *Nexmif* non-mutant (n = 3) versus mutant mice (n = 3) at postnatal day 21. (**D**, **E**) Area under the curve for glucose levels and determination of insulin levels after intra-peritoneal glucose tolerance tests in control C57BL6 mice outside the colony at 12 weeks (n = 3) and 40 weeks (n = 3), *Nexmif* non-mutant mice at 12 weeks (n = 8) and 40 weeks (n = 13), and *Nexmif* mutant mice at 12 weeks (n = 12) and 40 weeks (n = 18). Only male mice were used. Error bars indicate the standard deviation and “n” is the sample size. Significance was determined using 2-way ANOVA for repeated measurements followed by a multiple comparisons post hoc test. *Control vs. *Nexmif* mutant, P ≤ 0.05; ***Control vs. *Nexmif* mutant, P ≤ 0.001; ^$$^*Nexmif* non-mutant vs. *Nexmif* mutant, *P* ≤ 0.01; ^*#*^*Control* vs. *Nexmif* non-mutant, P ≤ 0.05. Ns: non-significant. The quantitative insulin sensitivity check index (QUICKI) was calculated with the following formula: 1/(log(fasting insulin μU/mL) + log(fasting glucose mg/dL))^17^, the HOMA B% with (20 × Insulin μU/mL)/(glucose (mmol/l) – 3.5)^16,66^. Significance was determined using one-way ANOVA analysis. Error bars indicate the standard deviation.

As Nexmif protein was mainly expressed in proliferating INS-1E cells, we determined if the loss of Nexmif influenced the percentage of beta cells that were proliferating in the pancreatic islets. Significantly lower percentages of proliferating beta cells were observed in the *Nexmif* mutant islets (Fig. 2A). Islets tended to be smaller and to contain fewer beta cells per islet in *Nexmif* mutant mice (Fig. 2B). This was confirmed in our islet distribution analysis, where islets containing over 200 beta cells were found only in *Nexmif* non-mutant mice (Fig. 2C). We measured a significantly decreased beta-cell surface (insulin positive cells, %) over total pancreas area in pancreatic sections from mutant and non-mutant *Nexmif* mice (*P* = 0.0423), suggesting a smaller total beta-cell mass (Fig. S2A). The body weight was unchanged (Fig. S2B). The percentage of beta cells undergoing apoptosis was similar in non-mutant and mutant *Nexmif* mice (Fig. S2C). We performed intraperitoneal glucose tolerance tests (ipGTT) at postnatal weeks 12 and 40. Randomization was done before the ipGTT by a technician so the experimenter was not aware of the genotypes of the mice. The technician revealed the group allocation for data analysis. The area under the curve was significantly higher in *Nexmif* mutant mice compared with control C57B6 mice outside of the colony at 40 weeks of age (Fig. 2D), while the difference was not significant versus *Nexmif* mutant mice of the same age. *Nexmif* mutant mice show however significantly increased glycemia levels at 15 min during the ipGTT in comparison to Nexmif non-mutant mice. In mutant *Nexmif* mice, both the insulin levels and the HOMA B%, reflecting beta-cell function, tended to be lower in comparison with the non-mutants (Fig. 2C)^16^.To exclude an alteration in insulin sensitivity, we calculated the quantitative insulin sensitivity check index (QUICKI)^17^ and found no statistical difference between the control, mutant, and non-mutant mice (*P* = 0.35) (Fig. 2E). To confirm the effect of Nexmif loss on beta cells in another species, we performed knockdown experiments in zebrafish. Nexmif knockdown led to a significant decrease in the beta-cell count as well as in the glucose-induced beta-cell expansion rate (Additional File 4: Fig. S3).

### Loss of Nexmif results in an increased number of DNA double-strand breaks

**Figure 3:**
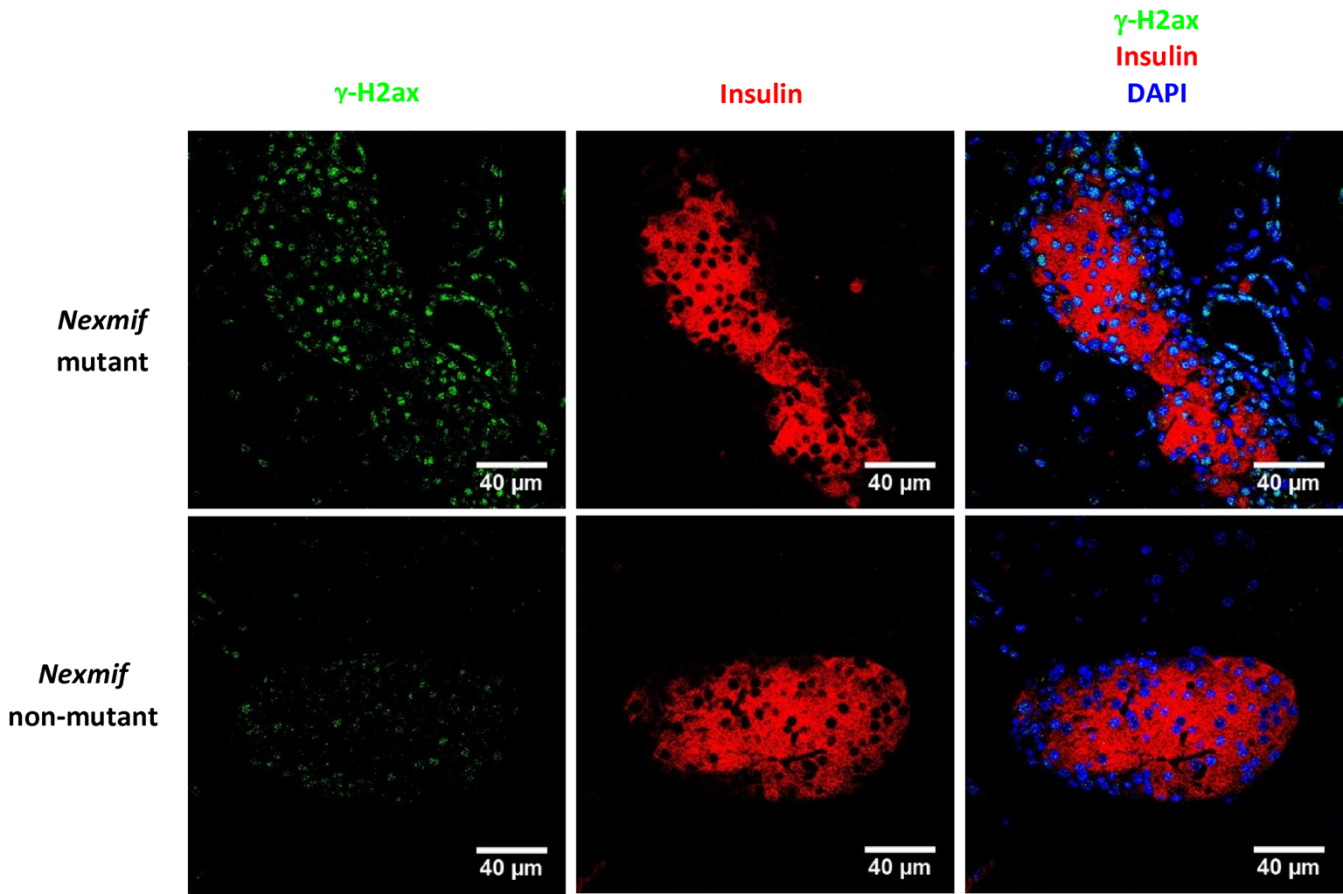
DNA damage in pancreatic islets of *Nexmif* mutant mice. DNA double-strand breaks marked by immunofluorescent staining with γ-H2AX (green) in pancreatic islets of *Nexmif* non-mutant (n = 3) and mutant mice (n = 3) at postnatal day 21. Insulin (red), DAPI (blue).

To determine if *Nexmif* could play a role in maintaining genomic stability, DNA double-strand breaks in *Nexmif* mutant and non-mutant pancreas were documented using the marker γ-H2AX^18^. There were more DNA double-strand breaks observed in *Nexmif* mutant than in non-mutant pancreatic islets (55% of insulin-positive cells versus 27%; Fig. 3).

### Loss of *Nexmif* in pancreatic islets results in the downregulation of genes involved in stress response

**Figure 4:**
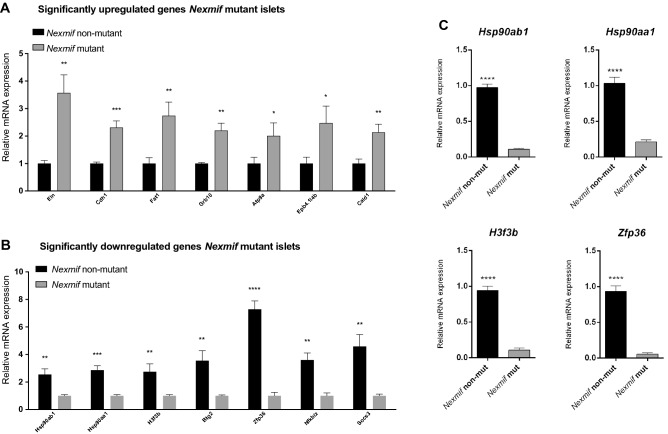
RNAseq in pancreatic islets of *Nexmif* mutant mice. (**A**) Gene expression profile of differentially expressed genes in pancreatic islets of male *Nexmif* mutant and *Nexmif* non-mutant mice. Kendall’s Tau was used for clustering possibly co-expressed pathway genes (n = 3). RNAseq mRNA expression data from pancreatic islets. The most highly expressed genes that were upregulated at least twofold in *Nexmif* mutant islets compared with *Nexmif* non-mutant islets (n = 3) are shown. (**B**) RNAseq mRNA expression data from pancreatic islets. The most highly expressed genes that were downregulated at least twofold in *Nexmif* mutant islets compared with *Nexmif* non-mutant islets (n = 3) are shown. (**C**) Confirmation of downregulated genes by qPCR: Hsp90ab1, Hsp90aa1, H3f3b, Zfp36 (n = 3). Error bars indicate the standard deviation. Two-tailed unpaired Student’s t-test: *P ≤ 0.05, **P ≤ 0.01. ***P ≤ 0.001, ****P ≤ 0.0001.

RNAseq analysis of *Nexmif* mutant and control islets revealed that genes upregulated (n = 711) in the mutant islets (Fig. S4) are mainly involved in developmental processes, cell migration, or cytoskeleton organization (Gene Ontology, Biological process, Table S1). Genes that were downregulated (n = 2418) in the *Nexmif* mutant islets (Fig. 4B) are mainly related to stress response, protein phosphorylation and folding, regulation of molecular function, and metabolic processes (Gene Ontology, Biological process, Table S1); among them are *Hsp90aa1*, *Hsp90ab1*, *H3f3b*, *and Zfp36* (Fig. 4B). Heat shock proteins are expressed in response to stress conditions. *H3f3b* encodes histone H3.3, which plays a role in beta-cell proliferation, while its main function is the maintenance of genomic integrity^19,20^. A big difference in expression was observed with *Zfp36*, which encodes a destabilizer of AU-rich element (ARE) mRNAs. Downregulation of *Hsp90aa1*, *Hsp90ab1*, *H3f3b*, and *Zfp36* was confirmed by qRT-PCR (Fig. 4C).

### *Nexmif* is highly expressed in response to oxidative stress

**Figure 5:**
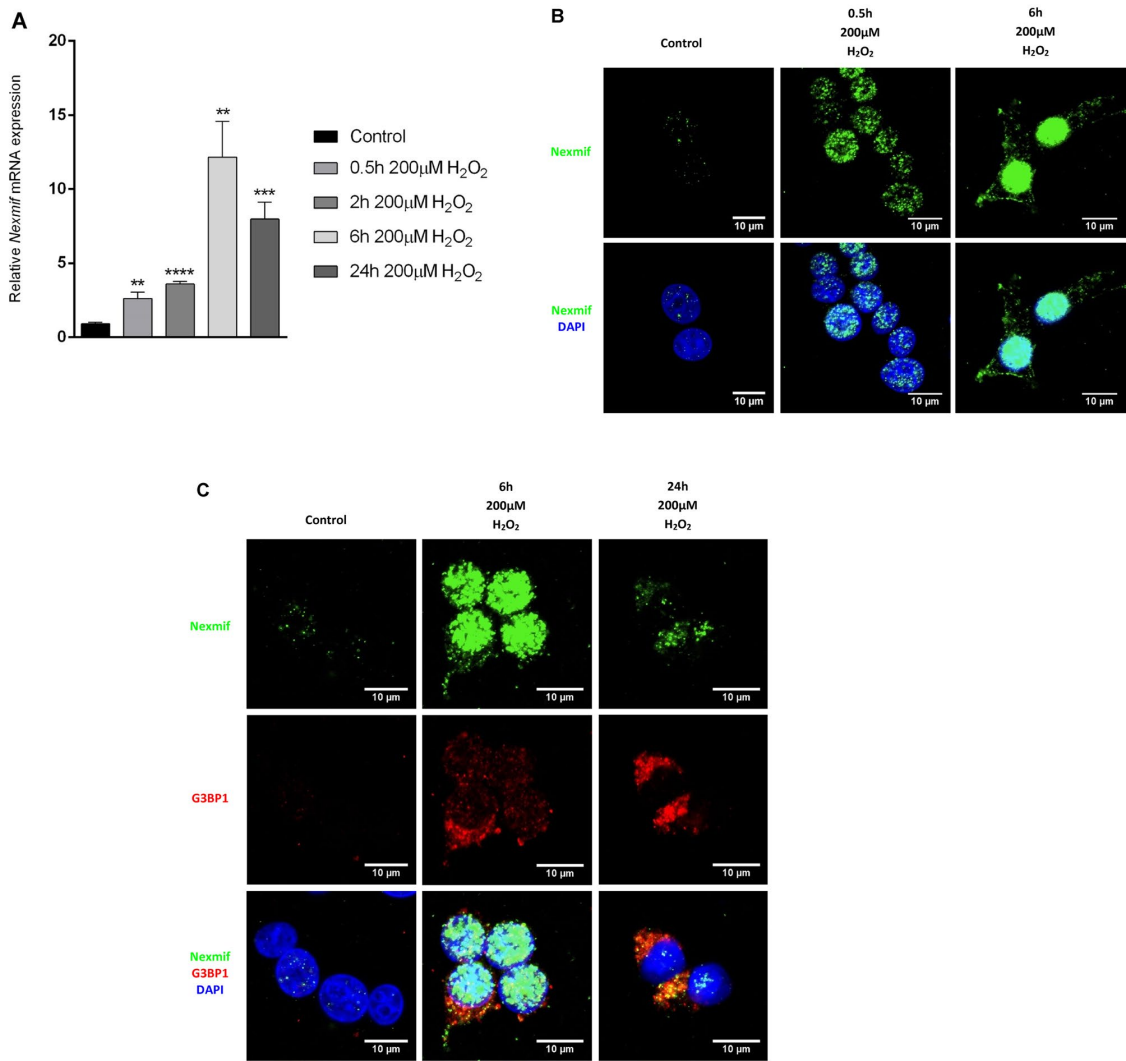
*Nexmif* expression in reaction to oxidative stress in INS-1E cells. (**A**) *Nexmif* mRNA expression over a time course in response to 200 µM H_2_O_2_ at time points 0 h, 0.5 h, 2 h, 6 h, and 24 h (n = 3). Error bars indicate the standard deviation. Two-tailed unpaired Student t-test: *P ≤ 0.05, **P ≤ 0.01, ***P ≤ 0.001, ****P ≤ 0.0001. (**B**) Nexmif localization (green) in INS-1E cells challenged with 200 µM H_2_O_2_ over a time course of 0 h, 0.5 h, and 6 h by immunofluorescence. DAPI (blue). (**C**) Expression and co-localization of Nexmif (green) by fluorescent in situ RNA hybridization (FISH) with G3BP1 (red) in INS-1E cells, marking stress granules by immunofluorescence.

Given the downregulation of stress-response genes in the *Nexmif* mutant islets, we examined *Nexmif* expression in response to cellular stress by exposing INS-1E cells to 200 µM H_2_O_2_ over a time course. *Nexmif* expression was strongly increased at both the mRNA and protein levels, with maximum expression after 6 h (Fig. 5A,B). The observed cytoplasmic localization of Nexmif with a granular pattern after H_2_O_2_ treatment indicated that Nexmif could be localized in stress granules or P-bodies in the cytoplasm, in particular a few hours after the oxidative stress. Co-staining with the stress granule marker GTPase-activating protein SH3 domain–binding protein 1 (G3BP1) after H_2_O_2_ treatment suggested that Nexmif localizes partially to stress granules (Fig. 5C).

### *Nexmif* is transcribed to form highly stable ARE mRNAs

One of the most strongly downregulated genes, *Zfp36*, is known as an important regulator of ARE mRNAs. ARE mRNAs are highly expressed by genes enriched with adenylate-uridylate-rich (AUUUA-elements) in their 3′ UTR. Zfp36 binds to these specific elements and plays an important role in their degradation. These elements are often found in proto-oncogenes, neuronally expressed genes such as *Nexmif*, and genes encoding cytokines and nuclear transcription factors^21^. In patients with *Nexmif* duplications, downregulation of gene expression has been reported^22^. This indicates that Nexmif might inhibit its own gene expression, potentially via the upregulation of *Zfp36*. It was determined that human *NEXMIF* contains 23 ARE sites in its 3′ UTR. Mouse *Nexmif* contains 15 ARE sites (AREsite, nibiru.tbi.univie.ac.at), a relatively high number. This indicates that *Nexmif* could be transcribed into ARE mRNAs.

**Figure 6:**
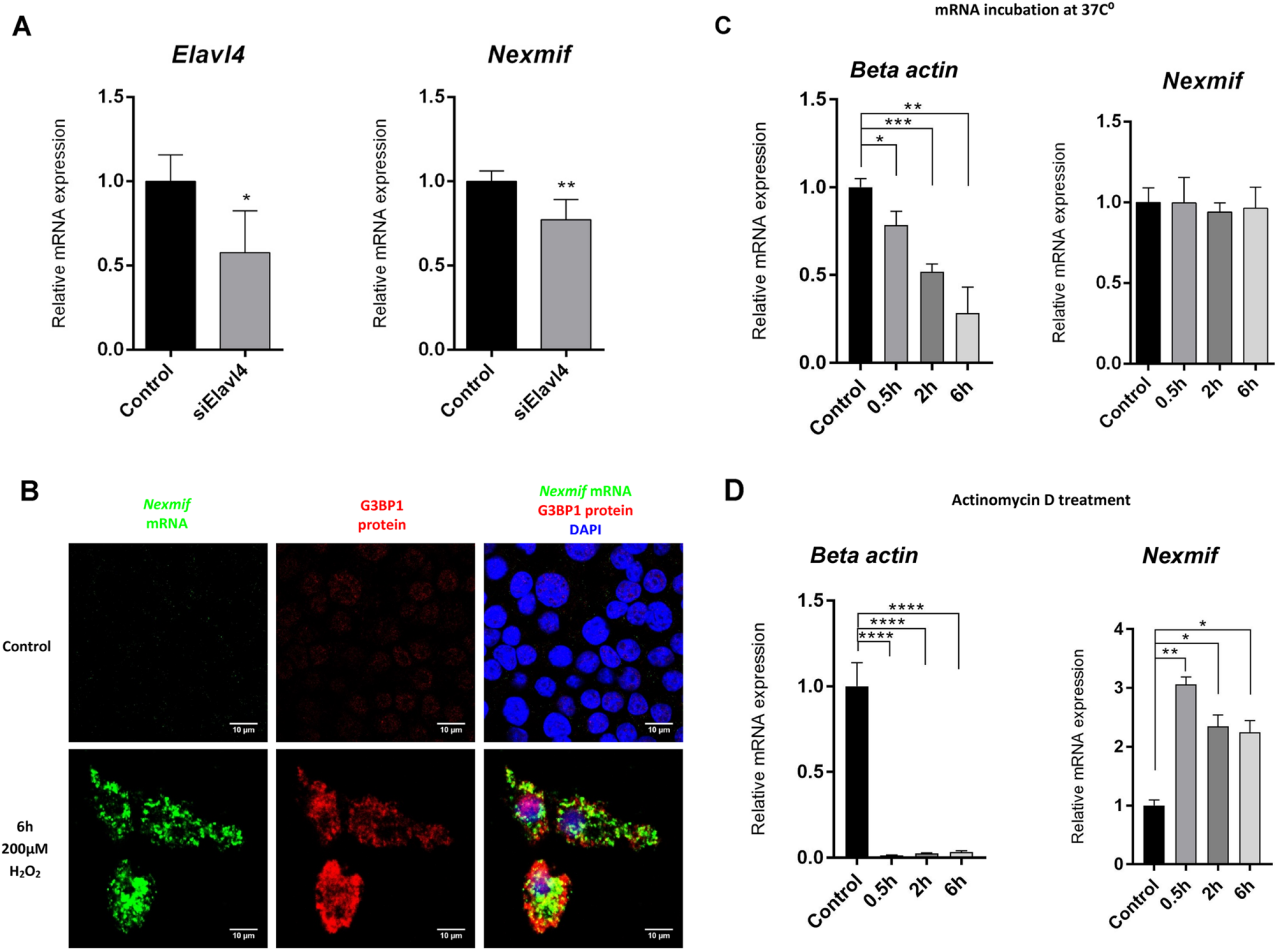
Determination of whether *Nexmif* transcripts are ARE mRNAs. (**A**) *Elavl4* and *Nexmif* expression in *Elavl4* knockdown INS-1E cells (n = 3). (**B**) Localization of *Nexmif* mRNA (green) and G3BP1 protein (red) after 6-h H_2_O_2_ treatment of INS-1E cells. (**C**) *Beta-actin* and *Nexmif* expression in INS-1E cells after incubation at 37 °C for 0 h, 0.5 h, 2 h and 6 h (n = 3). (**D**) *Beta-actin* and *Nexmif* expression in INS-1E cells after incubation with 10 µg/mL actinomycin D for 0 h, 0.5 h, 2 h and 6 h (n = 3). Error bars, standard deviation; n, sample size.

To determine if *Nexmif* is transcribed into ARE mRNAs, *Nexmif* expression was determined in *Elavl4* knockdown INS-1E cells*.* Elavl4 is a known stabilizer of ARE mRNAs^23^. Knockdown *of Elavl4* resulted in significantly reduced expression of *Nexmif* in INS-1E cells (Fig. 6A). To further investigate how *Nexmif* is transcribed into ARE mRNAs, the localization of *Nexmif* mRNA was determined after cells were exposed to an oxidative stress. ARE mRNAs are known to localize to stress granules, where they are protected under stressful situations. This was indeed observed for *Nexmif* mRNA after oxidative stress in INS-1E cells (Fig. 6B). Additionally, to determine *Nexmif* mRNA stability, we performed mRNA degradation tests in INS-1E cells. *Nexmif* mRNA expression remained stable, while beta-actin mRNA degraded gradually when incubated at 37 °C over a time course (Fig. 6C). Actinomycin D treatment was applied to inhibit transcription. Nexmif mRNA levels increased, while beta-actin mRNA levels declined sharply within 30 min (Fig. 6D).

### Loss of Nexmif induces phenotypic heterogeneity

Among *Nexmif* mutant mice, the first founder male did not show any abnormalities. In the second generation, one heterozygous mutant female was severely growth retarded, with low-set ears and a curved spine, and died at the age of 5 weeks (Additional File 6: Fig. S5A). From the third generation onward, more abnormalities were observed. Growth-retarded pups were observed among both male and female *Nexmif* mutant and non-mutant mice. Whole litters were regularly born dead, and we observed 4–6-week-old juveniles having seizures resulting in death. Other but less frequent observations were hind-limb polydactyly (Additional File 6: Fig. S5B) and microphthalmia (Additional File 6: Fig. S5C).

Another interesting observation was a female mouse missing part of or a whole X-chromosome. Mutant mice were identified by a 50-nt deletion in the DNA sequence of the *Nexmif* gene. A heterozygous female was crossed with a wild-type C57BL6 male. As the male passes on its intact X chromosome to the female progeny, all female offspring should carry a non-mutant *Nexmif* allele. However, one female carried only a mutated allele. The Y-chromosomal gene *Sry* was not amplified by PCR, indicating that it was not a case of sex reversal. The copy number of the X-linked genes in this female was similar to that in the male control, while being half that seen in the female control (Additional File 6: Fig. S5D). Mortality rates were higher in offspring of hemizygous males carrying the *Nexmif* mutation than in offspring of heterozygous females carrying the *Nexmif* mutation. Overall, each successive generation's phenotypes appeared to be more severe (Additional File 7: Fig. S6).

### Loss of Nexmif results in the activation of LINE1 elements

**Figure 7:**
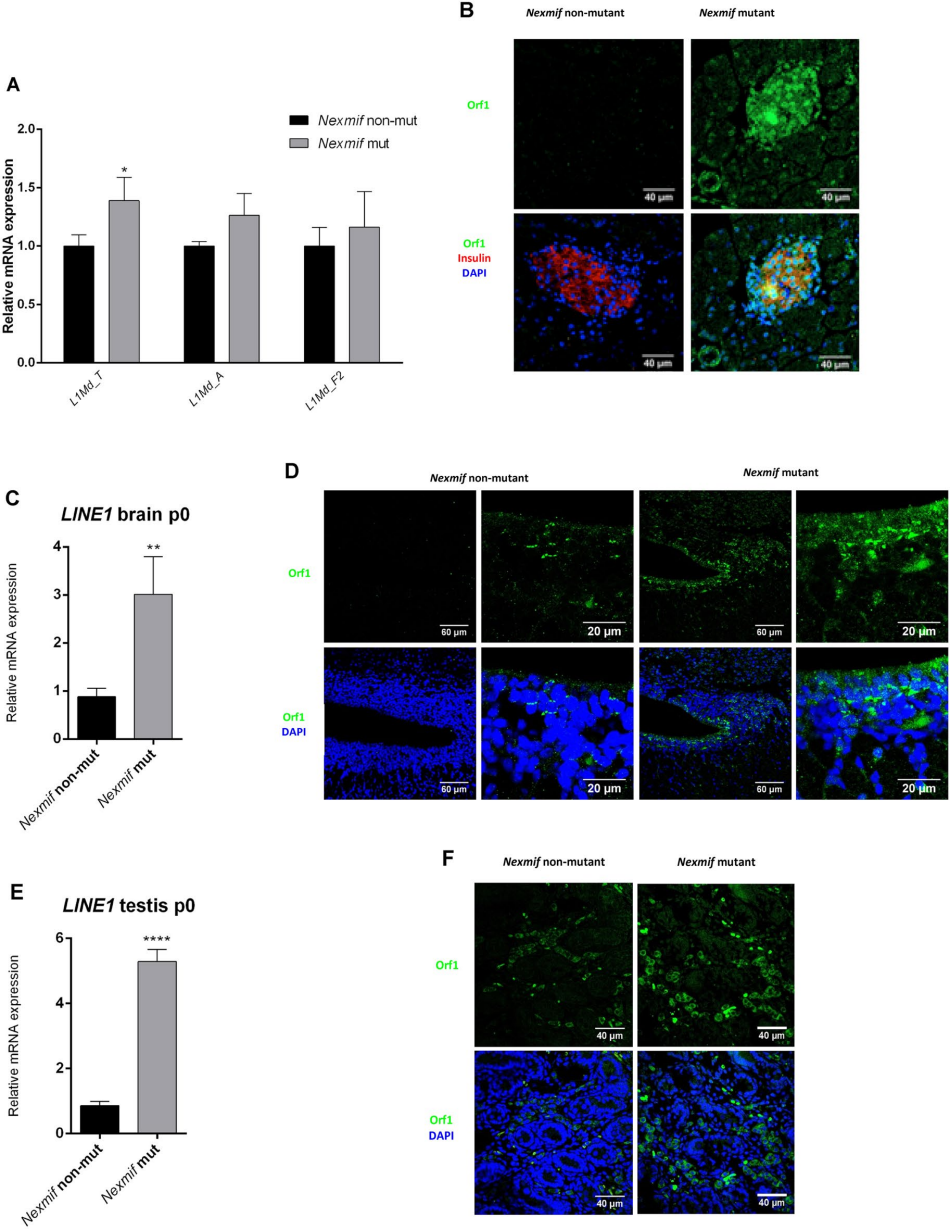
LINE1 activation. (**A**) RNAseq expression data for transposable LINE1 elements in pancreatic islets of 6-week-old *Nexmif* mutant and non-mutant mice (n = 3). (**B**) ORF1 protein (green) expression in *Nexmif* mutant and non-mutant mouse pancreas at postnatal day 21 by immunofluorescence. Insulin (red), DAPI (blue). (**C**) LINE1 mRNA expression in *Nexmif* mutant and non-mutant mouse brain at postnatal day 0 (n = 3). (**D**) ORF1 expression (green) in *Nexmif* mutant and non-mutant mouse brain (cortex) at postnatal day 0 by immunofluorescence. DAPI (blue). (**E**) LINE1 mRNA expression in *Nexmif* mutant and non-mutant mouse testis at postnatal day 0 (n = 3). ****P ≤ 0.0001. (**F**) ORF1 expression (green) in *Nexmif* mutant and non-mutant mouse testis at postnatal day 0 by immunofluorescence. DAPI (blue).

The expression of repeats was analyzed in the RNAseq results. In control pancreatic islets, the most abundant repeats were RNAs representing the LINE1 elements L1Md_T, L1Md_A, and L1Md_F2. In *Nexmif* mutant islets, expression of L1Md_T was significantly higher, while L1Md_A and L1Md_F2 showed a tendency to be upregulated (Fig. 7A). These LINE1 elements encode ORF1 protein. At postnatal day 21, ORF1 protein was highly expressed in *Nexmif* mutant pancreas and hardly detectable in non-mutant (Fig. 7B). As LINE1 is known to be strongly expressed in neural progenitor cells during brain development and in primordial germ cells in the testis^24^, we determined its expression in the brain and testis. LINE1 expression was significantly higher in the mutant brain at postnatal day 0 (Fig. 7C,D). A similar observation was made for the testis (Fig. 7E,F).

## Discussion

This study started with the diagnosis of non-autoimmune diabetes and mental retardation in a 5-year old boy with a truncated *NEXMIF* variant. During our study, we identified a female with non-autoimmune diabetes and learning disabilities who was carrying a *NEXMIF* variant, underlining a role for NEXMIF in the development of diabetes. At the same time, more individuals with mental retardation and *NEXMIF* variants were identified^25^. From these patients, one additional female has been described to have diabetes, suggesting that *NEXMIF* plays a role in diabetes development. We hypothesize that an inadequate beta-cell mass prompted diabetes in both patients, as insufficient beta-cell mass is key in the pathogenesis of diabetes. Indeed, we demonstrated both RNA and protein NEXMIF/Nexmif expression in pancreatic beta cells. The nuclear and cytoplasmic localization we observed in beta cells has been described in brain neurons by Ishikawa et al.^3^. The metabolic microenvironment could influence the localization of the protein, as has been observed of the beta-cell transcription factor Pdx1^26,27^.

The RNAseq data show decreased expression of *H3f3b*, encoding H3.3. Proper deposition of histone H3.3 has been shown to play a role in human beta-cell proliferation and could be a cause of reduced beta-cell proliferation in *Nexmif* mutant islets^19^.

One of the most strongly downregulated gene in the mutant islets was *Zfp36*. As Zfp36 is an ARE-binding factor, we tested whether Zpf36 might affect Nexmif itself, as Nexmif could potentially repress its own expression^22^ through the upregulation of *Zfp36*. The presence of a significant number of ARE sites in the *Nexmif* 3′ UTR, plus the fact that Nexmif localizes to stress granules after oxidative stress, makes this hypothesis plausible. RNA-binding proteins and mRNA processing are emerging mechanisms regulating beta-cell function and survival.

One important observation is that the patients described in the literature have a highly variable phenotype. They are all mentally retarded, but the associated phenotype differs, even among family members with the same mutation. Additionally, females carrying *NEXMIF* mutations can be either severely mentally retarded or unaffected. This does not seem to be due to X-inactivation, which has been shown to be random in both patients and unaffected carriers. Unaffected female carriers are known to inherit *NEXMIF* mutations maternally, while affected females acquire it de novo, indicating a parent-of-origin effect. Previous work on the silencing of *Nexmif* in the developing mouse brain indicated that Nexmif plays a role in neuronal migration and dendritic growth^28^. Increased cell surface expression of N-cadherin as well as altered delta-catenin signaling and actin dynamics explain the impaired neuronal morphogenesis at the molecular level. In addition, knockdown experiments in neuronal cells have also shown upregulation of beta-integrin, which is implicated in enhanced cell–cell adhesion^29^.

We studied a mouse model carrying a truncated *Nexmif* variant. Interestingly, we observed high mortality and high phenotypic variability between siblings, independent of the *Nexmif* mutation. The mortality rate and phenotypic variability seemed to increase with each generation, and even more severely when the carrier parent was a male rather than a female. This supports the notion of a parent-of-origin effect, as indicated by observations in *NEXMIF* families. Other phenotypes shared between our mouse model and *NEXMIF* patients are growth retardation, obesity, and seizures.

Our study indicates that the observed variability could be due to a loss of genomic integrity. We observed an increase in DNA double-strand breaks in the pancreas of *Nexmif* mutant mice. Additionally, RNAseq on pancreatic islets indicated a downregulation of genes involved in the stress response. Interestingly, *Hsp90*, which was found to be downregulated in *Nexmif* mutant islets, plays an important role in neurite outgrowth and stress response. Most importantly, *Hsp90* has also been identified as an evolutionary capacitor, allowing a population to accrue genetic variation when insufficiently expressed. Lack of *Hsp90* expression results in phenotypic variation in the offspring of *Drosophila* and cave fish, including offspring that re-express *Hsp90*^30,31^. This is interesting, as we observed high variation in our mouse *Nexmif* colony, including in offspring carrying the non-mutant *Nexmif* allele. Several studies have reported that the activation of transposable elements plays a role in causing variation. In flies and mammals, Hsp90 has been shown to bind chromatin at gene regulatory elements, where it stabilizes transcriptional and epigenetic factors^32–35^. Additionally, in *Drosophila* a lack of Hsp90 results in the transposition of transposable elements (TEs), possibly disrupting important genes. In *Drosophila* and mice, Hsp90 has been shown to control the transposition of transposable LINE1 elements in the male germ line via piRNA pathways^36,37^. Another downregulated gene that is known to play an important role in maintaining genomic integrity is *H3f3b*. *H3f3b* and *H3f3a* both encode the highly conserved protein, histone H3.3. Lack of histone H3.3 induces dysfunction of heterochromatin structures at pericentric regions and telomeres, resulting in mitotic and meiotic defects^38–40^. It is likely that H3f3b (H3.3) regulates LINE1 elements, as H3.3 deposited in heterochromatic genomic regions over LINE1 elements represses LINE1^19,20,41^. A decrease in H3.3, as shown here in *Nexmif* mutant mice, leads to an increase in LINE1 element expression. The observation of a female missing a part or all of one X chromosome suggests that the loss of Nexmif can result in meiotic defects, which could be due to the downregulation of *H3f3b*. Histone H3.3 has also been shown in embryonic stem cells to be essential for silencing transposons, among which LINE1 elements cause a loss of genomic integrity^41^. Additionally, histone 3.3 has been shown to be essential for proper neuronal differentiation, neurite outgrowth, and proliferation^42^. Histone H3.3 deposition occurs during development and also after UV-induced DNA damage^43,44^. The downregulation of *Hf3bf* in *Nexmif* mutant mice as well as the importance of histone H3.3 deposition during brain development and DNA damage response indicate that there might be an important link between histone H3.3 and Nexmif. As previously mentioned, Nexmif was suggested to have a similar function as Atrx^3^. Interestingly, Atrx has been shown to bind and control histone H3.3 deposition at pericentric regions, telomeres, and silenced methylated regions to maintain epigenetic modifications such as H3K9me3^39,45^. Another parallel with Atrx is the observation of polydactyly in one mouse of a colony lacking *Atrx* expression in chondrocytes. Polydactyly did not seem to be a direct effect of the loss of *Atrx*, as other mice in the colony were not affected^46^. Polydactyly is rare and the mouse we identified in our colony was not a carrier of the *Nexmif* mutation, indicating an indirect effect. Loss of *Nexmif* expression might not only regulate histone H3.3 expression but also H3.3 deposition^44,46^. A possible physical association of Nexmif with histone H3.3 should be determined in future studies.

Both Hsp90 and histone H3.3 have been implicated in LINE1 inhibition^41^. We observed increased LINE1 expression in the pancreas, brain, and testis of *Nexmif* mutant mice. LINE1 activity could be detrimental to genomic integrity, as the LINE1 mRNA encodes two proteins (ORF1 and ORF2) with endonuclease and retro-transcriptase activity. These two proteins bind to the LINE1 mRNA, create double-strand breaks in DNA, and aid in the retrotransposition of LINE1 back into the genome. LINE1 elements play a major role in evolution and variability in a population due to their ability to retrotranspose in the germ line^47–51^. Additionally, they have been shown to play a major role in regulating chromatin structure and accessibility during development and to regulate the expression of many other genes^52^. LINE1 elements have been shown to be highly active in neural progenitors, but also in the male germ line. Increased LINE1 activity is associated with oxidative stress, autism, schizophrenia, Alzheimer’s disease, auto-immune diseases, ataxia telangiectasia and Rett syndrome^53–58^. Rett syndrome patients have characteristics similar to those of *NEXMIF* patients. The mutated gene in Rett syndrome, MECP2, is also involved neurite outgrowth and LINE1 inhibition^54^. These observations and our results could imply a similar role for NEXMIF.

## Methods

### Genetic analysis

Exomic regions of patient and parent DNA were selected with liquid phase capture (Sureselect XT, Agilent, Santa Clara, CA, USA) and analyzed with massive parallel sequencing in an Illumina HiSeq2000 high-throughput sequencer, using the manufacturer's protocols. We considered only exonic and splicing variants (± 10 bp from the intron–exon boundary) for further study. We excluded synonymous variants with a minimum allele frequency ≥ 0.01 in the dbSNP database. Remaining variants were checked in the Exome Variant Server, ExAC, and gnomAD databases*.* The pathogenic *NEXMIF* variant was confirmed with Sanger sequencing.

We excluded all variants inherited from either parent in the trio under the hypothesis of de novo mutations as the underlying cause of the syndromic diabetes. The variant was confirmed by Sanger sequencing. The absence of *NEXMIF*-truncating variants in the gnomAD (https://gnomad.broadinstitute.org/) is consistent with a genetic disease due to NEXMIF loss of function.

Raw sequencing data were analyzed using a locally developed pipeline integrating BWA, SAM, PINDEL, and ANNOVAR algorithms for alignment on the hg19 human reference genome and the identification and annotation of variants, including the interrogation of databases for known polymorphisms and mutations^59^. The most prominent variants were selected according to the prediction score for damaging effects on the respective proteins using SIFT (score < 0.05), Polyphen-2 (score > 0.85), MutationTaster-2 (score > 0.5), the likelihood-ratio test LRT (score > 0.9), PhyloP (score > 0.85), and GERP (score > 2) and confirmed by Sanger sequencing. Variants with an allelic frequency ≥ 0.1% in the 1000 Genomes Project, Exome Variant Server database were filtered out.

### Cell culture

Clonal INS-1E cells (RRID:CVCL_0351, obtained from P. Maechler) were plated at a concentration of 2 × 10^5^ cells/mL in a 12-well plate (1 mL/well) and cultured for 72 h in a humidified atmosphere containing 5% CO_2_ in complete medium composed of GlutaMax RPMI-1640 (ThermoFisher Scientific 61870036) supplemented with 5% heat-inactivated fetal calf serum (Gibco 10500064), 1 mM sodium pyruvate, 50 μM β-mercaptoethanol (Gibco 21985023), 10 mM HEPES, 100 μg/mL streptomycin, and 100 U/mL penicillin^60^.

### Cell fixing and staining

Cells were cultured as described, in wells containing coverslips coated with poly-L-ornithine (Sigma, P4957). To fix the cells, the medium was aspirated and the coverslips were covered with 4% PFA for 6 min. Coverslips were washed three times with PBS and covered with a 3% BSA in PBS solution for 1 h. Primary antibodies were diluted in 3% BSA and slides were incubated at 4 °C overnight. Coverslips were washed 3 times with PBS and secondary antibodies in PBS (1:500) were added for 1 h at room temperature. Coverslips were washed 3 times with PBS and covered in DAPI in PBS (1:50,000, Invitrogen D1306) for 5 min. Coverslips were then washed 3 times with PBS. Mounting medium was applied to microscope slides and the inverted coverslips were placed on top. Slides were kept at 4 °C. The primary and secondary antibodies used and their dilutions are presented in Additional File 9: Table S2.

### Quantitative real-time PCR

RNA extraction was performed with a Qiagen RNAeasy mini kit (Qiagen 74136) using the manufacturer’s instructions. RNA (3 µg, 0.1 µg/µL) was reverse transcribed to cDNA using 0.5 µg random hexamers (Microsynth), 400 U Moloney murine leukemia virus reverse transcriptase (Invitrogen, 28025-013), 20 U recombinant RNasin (Promega, N251B), 2 mmol/L dNTPs (Rovalab, R203), and 40 mmol/L dithiothreitol (Invitrogen). Quantitative real-time PCR was performed with the Power SYBR Green PCR Master Mix (Applied Biosystems, 4367659) and using an ABI StepOne Plus Sequence Detection System (Applera Europe). Gene expression data were normalized using the housekeeping gene 18S RNA unless otherwise stated. qPCR primers used in this study can be found in Additional File 10: Table S3.

cDNA from INS-1E cells after knockdown of Elavl4 by siRNA was kindly provided by the laboratory of Prof D. Eizirik, ULB Center for Diabetes Research, Université Libre de Bruxelles, Belgium^61^.

### Tissue collection

Brain, pancreas, and testis were collected at postnatal days 0, 3, 7, 14, and 21 and/or 42. Tissue was snap frozen before RNA extraction or immersed in a 4% PFA solution overnight. Tissue was then transferred to PBS for 24 h and afterwards dehydrated and embedded in paraffin, and 5-μm sections were prepared. For analysis of proliferative cells, 5 sections at 50-μm intervals were selected from each pancreas.

### Immunofluorescent staining of tissue sections

Tissue slides were twice covered in Neoclear solution for 5 min. To rehydrate, slides were immersed in the following solutions: 100% ethanol, 100% ethanol, 95% ethanol, 70% ethanol, 50% ethanol, and 35% ethanol. Slides were washed three times in PBS. Next, slides were heated for 20 min in citrate buffer and washed three times in PBS. Slides were permeabilized with 0.3% Triton X-100 in PBS for 10 min. Blocking was performed with 2% BSA, 0.3% Triton X-100, and 5% goat serum for 30 min. Slides were incubated with 2% BSA, 1% goat serum, and the primary antibody of interest in PBS solution overnight. Slides were washed three times with PBS and incubated with the corresponding secondary antibody in PBS (1:1000) for 2 h at RT. Slides were then washed three times and incubated with DAPI in PBS (1:50,000). Next, slides were washed three times with PBS and mounted. Slides were analyzed by use of a Nikon A1r spectral confocal microscope. Slides used for determining the number of proliferating cells were scanned with a Zeiss Axio Scan.Z1 wide-field scanner. Antibodies used in this study are presented in Additional File 9: Table S2.

### H_2_O_2_ treatment INS-1E cells

INS-1E cells were cultured as described above. After culturing for 72 h, cells were treated with 200 µM H_2_O_2_ in order to induce a stress response^62^. Cells were collected after 30 min, 2 h, 6 h, and 24 h for RNA extraction and immunofluorescent staining.

### Generation of *Nexmif* mutant mice

We consulted different web resources to design the following top- and bottom-strand SgRNA oligos to target *Nexmif* specifically^63^, https://zlab.bio/guide-design-resources):

#### Sgrna1

- 5′ caccgAATGATCGGGTGCTTCAATC
- cTTACTAGCCCACGAAGTTAGcaaa 5′  ⇒ 5′
- aaacGATTGAAGCACCCGATCATTc.

#### Sgrna2

- 5′ caccgCACGGCTTCTTAGATGGCGG
- cGTGCCGAAGAATCTACCGCCcaaa 5′  ⇒ 5′
- aaacCCGCCATCTAAGAAGCCGTGc

2 µg of top- and bottom-strand oligos was added to a total volume of 50 µL Neb2 buffer. Samples were heated for 3 min at 95 °C and cooled down for 45 min at RT. Next, 5 µL of annealed oligos were diluted with 45 µL nuclease-free water and the concentration was determined by Nanodrop. Next, 5 µg of PX330 was digested with Bbsl in a total volume of 50 µL for 3 h at 37 °C. The digested PX330 was loaded on a 0.5% agarose gel and excised and purified with a gel extraction kit (Qiagen QIAquick, Qiagen 28,106, manufacturer’s protocol). A sample was run on a 2% agarose gel to verify the presence of purified PX330. Next, 1 µL annealed oligos (75–600 ng), 1.5 µL PX330 Bbsl vector (± 100 ng), 1 µL T4 ligase buffer, 1 µL T4 ligase enzyme, and 5.5 µL nuclease-free water were mixed. Samples were incubated at RT for 10 min and then at 65 °C for 10 min. Ligated PX330-SgRNA was used to transform Max Efficiency DH5α Competent Cells using the manufacturer's protocol (Invitrogen 18265017). We screened for the presence of PX330 by PCR, using PX330 primer 5′ GGCCTATTTCCCATGATTCC 3′ and the bottom-strand SgRNA oligo. Colonies were picked, put in the PCR mix, and incubated overnight in mini-culture at 37 °C. The PCR product was run on a 2% agarose gel, following the manufacturer’s instructions for the mini-preparation (Qiagen QIAprep Spin miniprep). PX330 plasmids containing SgRNA1 and SgRNA2 were selected and injected into DBA2 oocytes fertilized with C57BL6 sperm. We generated a genetically modified mouse line from a male with a truncating *Nexmif* variant p.Pro68* due to a 50-nucleotide deletion, as it would most likely be the most detrimental. The top 3 most likely off-target regions (https://crispr.cos.uni-heidelberg.de/) were confirmed by PCR and Sanger sequencing to be not affected. Mice were backcrossed with C57BL6 mice for at least 5 generations before being used in experiments. We used a total of 118 animals. The sample size was chosen according to standards in the field.

### Genotyping

Ear punches were collected and immersed in lysis buffer (Viagen Biotech 102-t) with proteinase K (Macherey Nagel 740506). Samples were incubated at 55 °C for 8 h and then at 85 °C for 1 h. Screening was performed using *Nexmif* primers: Primer F: TCTACCTTTTCCTGGGCTGA, Primer R: AGCAACCTAAGCAAGTCCTG. Accuprime (Life Technologies 12339024, manufacturer’s protocol) was used for the DNA amplification. The resulting product was 600 bp in length.

### Glucose intolerance measurements

Glucose (2 g/kg body weight) was administered intra-peritoneally in 6-h-fasted male mice before measurements of glucose levels. Glucose measurements (Accu-Check, Roche Diagnostics, Rotkreuz, Switzerland) and blood collection (Sarstedt low-bind microtubes) from the tail vein were performed at times 0 min, 15 min, and 30 min. Glucose levels were additionally measured at 60 min and 90 min.

### Insulin level measurements

Plasma insulin levels from blood sampled at 0 min, 15 min, and 30 min after glucose administration were determined using an ultrasensitive mouse insulin ELISA (Mercodia AB, Uppsala, Sweden).

### Islet isolation

Pancreatic islets were isolated from 6-week-old male mice, using collagenase P (Roche 11249002001) with a Histopaque (Sigma-Aldrich 10771) gradient as described previously^64^. After the gradient step, RNA was extracted from hand-picked islets.

### RNAseq

Gene expression in pancreatic islets from 6-week-old male *Nexmif* mutant and non-mutant mice was profiled with the Affymetrix Genechip system. Sequencing quality control was done with FastQC v.0.11.5. Reads were mapped with the TopHat v.0.2.1.1 software to the USCS mm10 reference genome. The table of counts with the number of reads mapping to each gene feature on UCSC mm10 was prepared with HTSeq v0.6p1. The differential expression analysis was performed with the statistical analysis R/Bioconductor package edgeR v. 3.14.0. Briefly, the counts were normalized according to the library size and filtered^65^. The genes having a count above 1 count per million reads (cpm) in at least 3 samples were kept for the analysis. Additionally, repeats were analyzed. Selected differentially expressed genes were verified by qPCR (at least twofold difference, with an SD < 25% of the average value).

### In situ hybridization

Samples were fixed with 4% paraformaldehyde for 6 min and then permeabilized in 0.3% Triton X-100 in PBS. After three rinses with PBS, the samples were rinsed 2 times with 1 × saline sodium citrate (SSC)/50% formamide, fluorescent probes were added, and FISH was performed overnight at 37 °C (5 µL of probes in 500 µL solution). FISH probes were DNA oligos labeled with a ULYSIS Alexa Fluor 546 Nucleic Acid Labeling Kit (following the manufacturer’s protocol).

Slides were washed three times with PBS and incubated for 30 min with 5% BSA PBS solution for blocking. Next, the slides were incubated for 2 h with G3BP1 antibody (1:200) in PBS, after which they were washed three times with PBS. The slides were then incubated for 1 h with secondary antibody (donkey anti-goat Alexa Fluor 488), washed three times with PBS, and incubated with DAPI for 5 min. Finally, the slides were washed three times with PBS and coverslipped with mounting medium.

### Actinomycin D treatment

INS-1E cells were cultured as described above. After being cultured for 72 h, cells were treated with 10 nM actinomycin D (Sigma, A1410). Cells were collected after 30 min, 2 h, and 6 h for RNA extraction.

### mRNA degradation

INS-1E cells were cultured as described above. After being cultured for 72 h, cells were collected and mRNA was extracted in a total volume of 45 µL. The extracted mRNA was redistributed in 10-µL aliquots to 4 Eppendorf tubes. The tubes were incubated at 37 °C for 0 h, 0.5 h, 2 h, and 6 h. RT-PCR was performed and the degradation of *Actb* (beta actin) and *Nexmif* mRNAs was determined by the Ct value.

### Zebrafish methods

See Additional File 1.

### Statistical analysis

Paired t-test analyses were performed with Prism GraphPad software (v.8.4.2) when appropriate. Data are presented as means ± standard error of the mean (SEM) and are significant at *P ≤ 0.05, **P ≤ 0.01, ***P ≤ 0.001, and ****P ≤ 0.0001.

### Ethics approval and consent to participate

We obtained informed consent to participate from all the participants or their legal guardians. The study was approved by the “Commission Centrale D’éthique de la Recherche sur L’être Humain” (#CER 11-140-2°).

All animal-based research was conducted at the University of Geneva Medical Center with the approval of the animal care and experimentation authorities of the Canton of Geneva (#GE/153/16).

We confirm that all methods were performed in accordance with the relevant guidelines and regulations.

We also confirm that the study is reported in accordance with ARRIVE guidelines.

### Consent for publication

We obtained consent for publication from the participants or their legal guardians.

### Web resources

GTExportal, (https://gtexportal.org/home/gene/KIAA2022, SIFT, http://sift.jcvi.org, PolyPhen2, http://genetics.bwh.harvard.edu/pph2/, Mutation Taster, http://www.mutationtaster.org, Likelihood-ratio test query, http://www.genetics.wustl.edu/jflab/lrt_query.html, GnomAD, https://gnomad.broadinstitute.org/, Zhang lab, https://zlab.bio/guide-design-resources, CCTop, https://crispr.cos.uni-heidelberg.de/.

## Supplementary Information

Supplementary Information 1.

Supplementary Table S1.

## Data Availability

The data generated or analyzed during this study are included in this article and its supplemental information files or will be available from the following repository: https://zenodo.org/.
