# Supplementary material for: Loss of Nexmif results in the expression of phenotypic variability and loss of genomic integrity
Source: Sci Rep. 2022 Aug 15;12:13815. doi: 10.1038/s41598-022-17845-1 (PMC9378738; doi:10.1038/s41598-022-17845-1)
Supplement: Supplementary file 1 — Supplementary Information 1. [file 41598_2022_17845_MOESM1_ESM.docx]

**Additional figures and files**

**D**

**FIGURE S1**

**
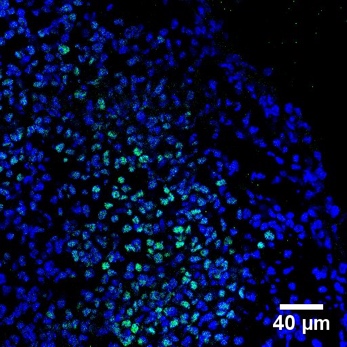

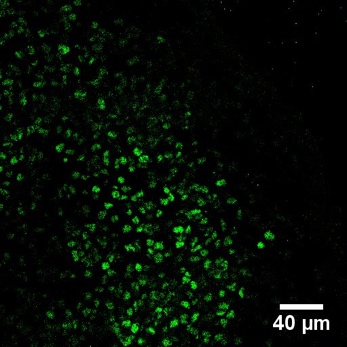

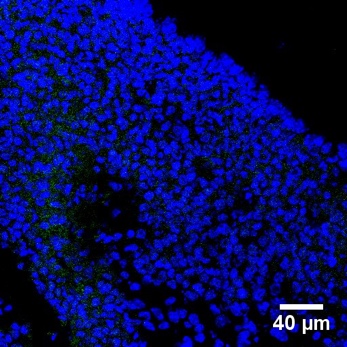

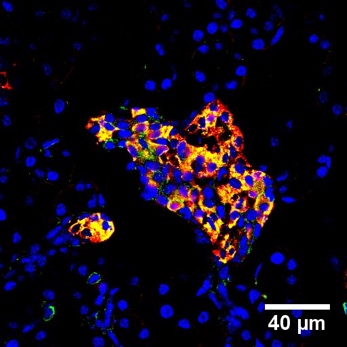

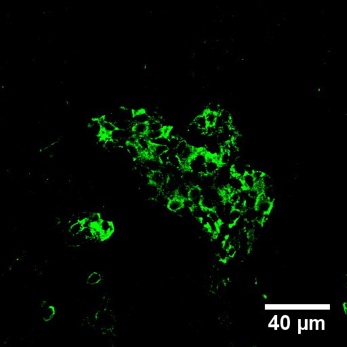
**

***Nexmif* mutant**

***Nexmif* non-mutant**

***Nexmif* mutant**

***Nexmif* non-mutant**

**Mouse pancreas postnatal day 0**

**Mouse brain postnatal day 0**

**Nexmif**

**Nexmif**

**Insulin**

**DAPI**

**
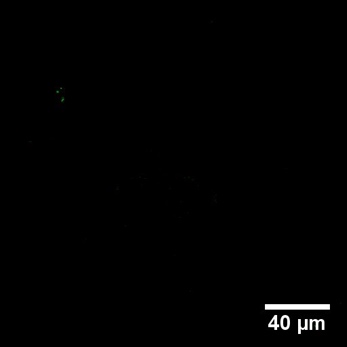

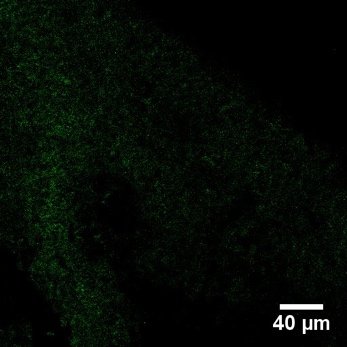

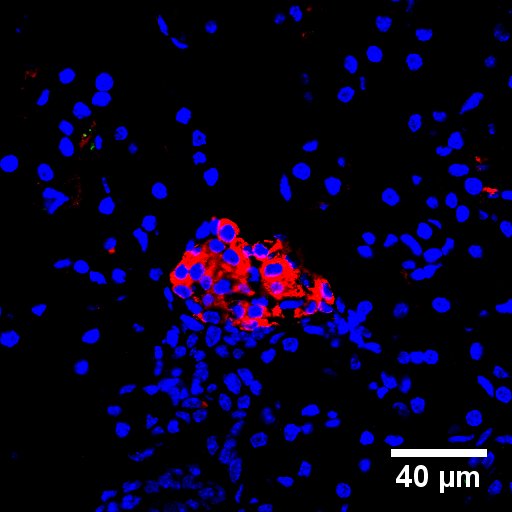
**

**FIGURE S2**

**FIGURE S3**

**B**

**A**

**FIGURE S4**

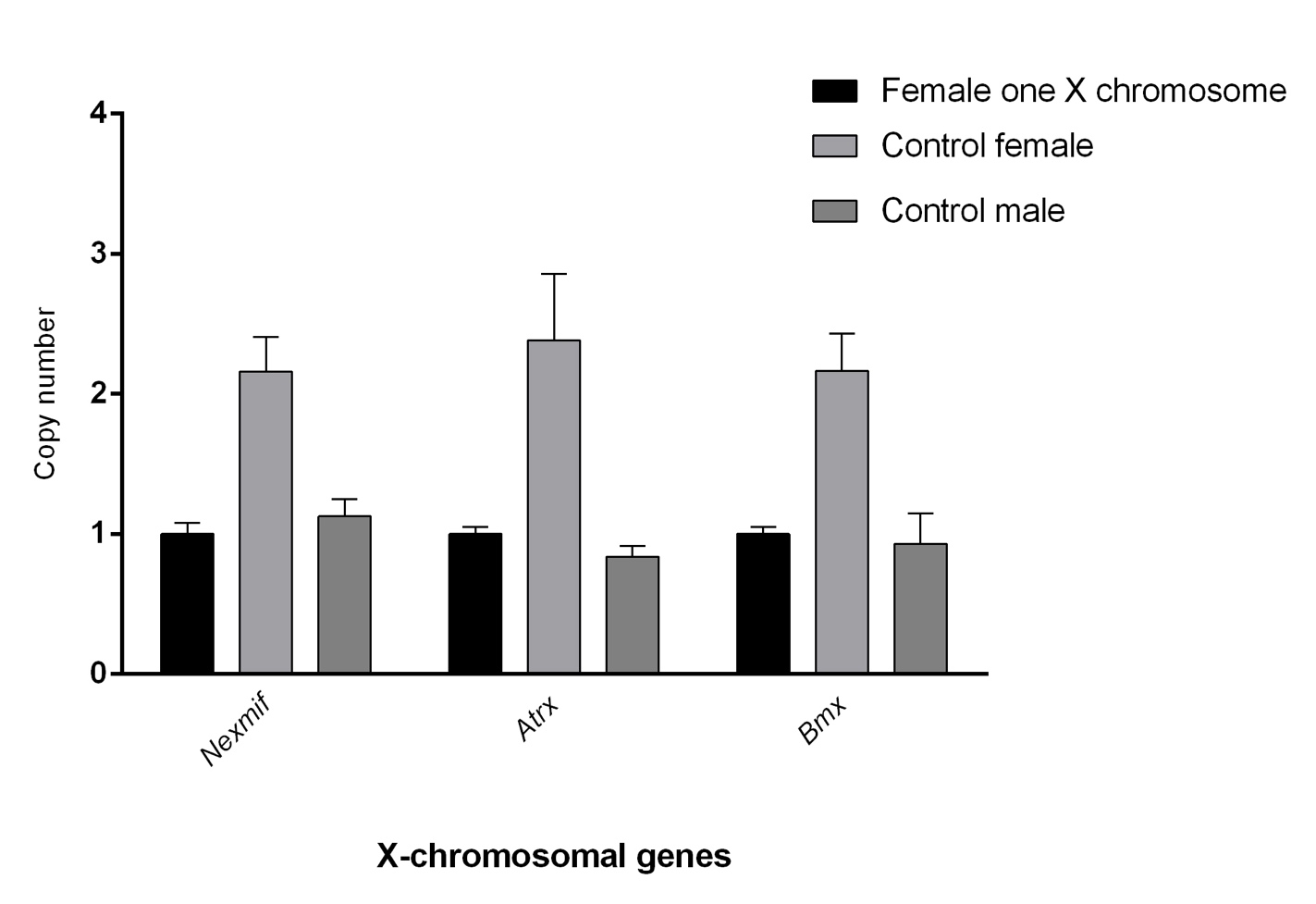
**FIGURE S5**

**A**

**B**

**C**


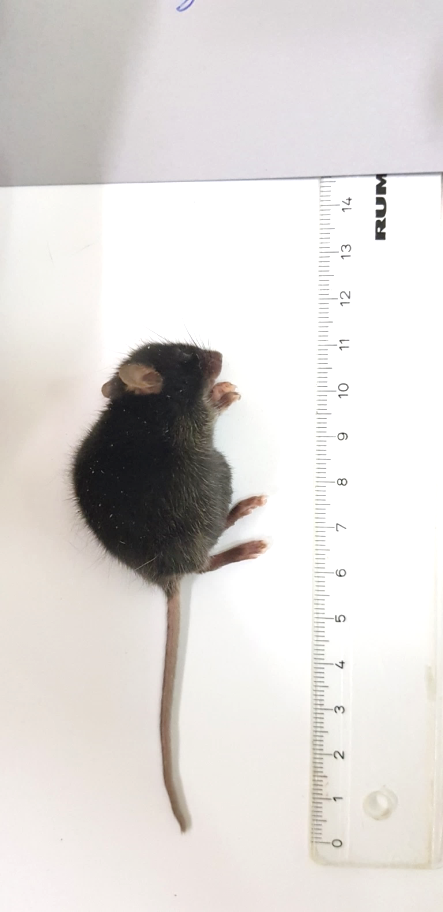

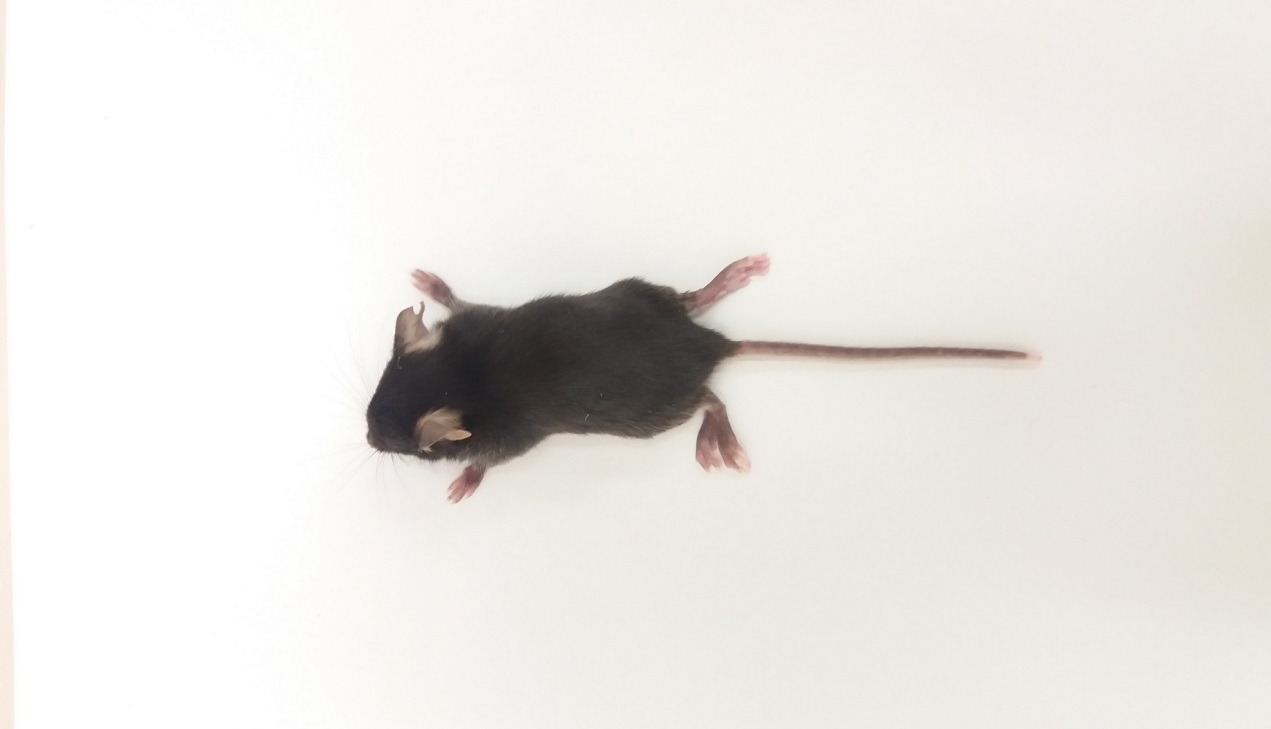

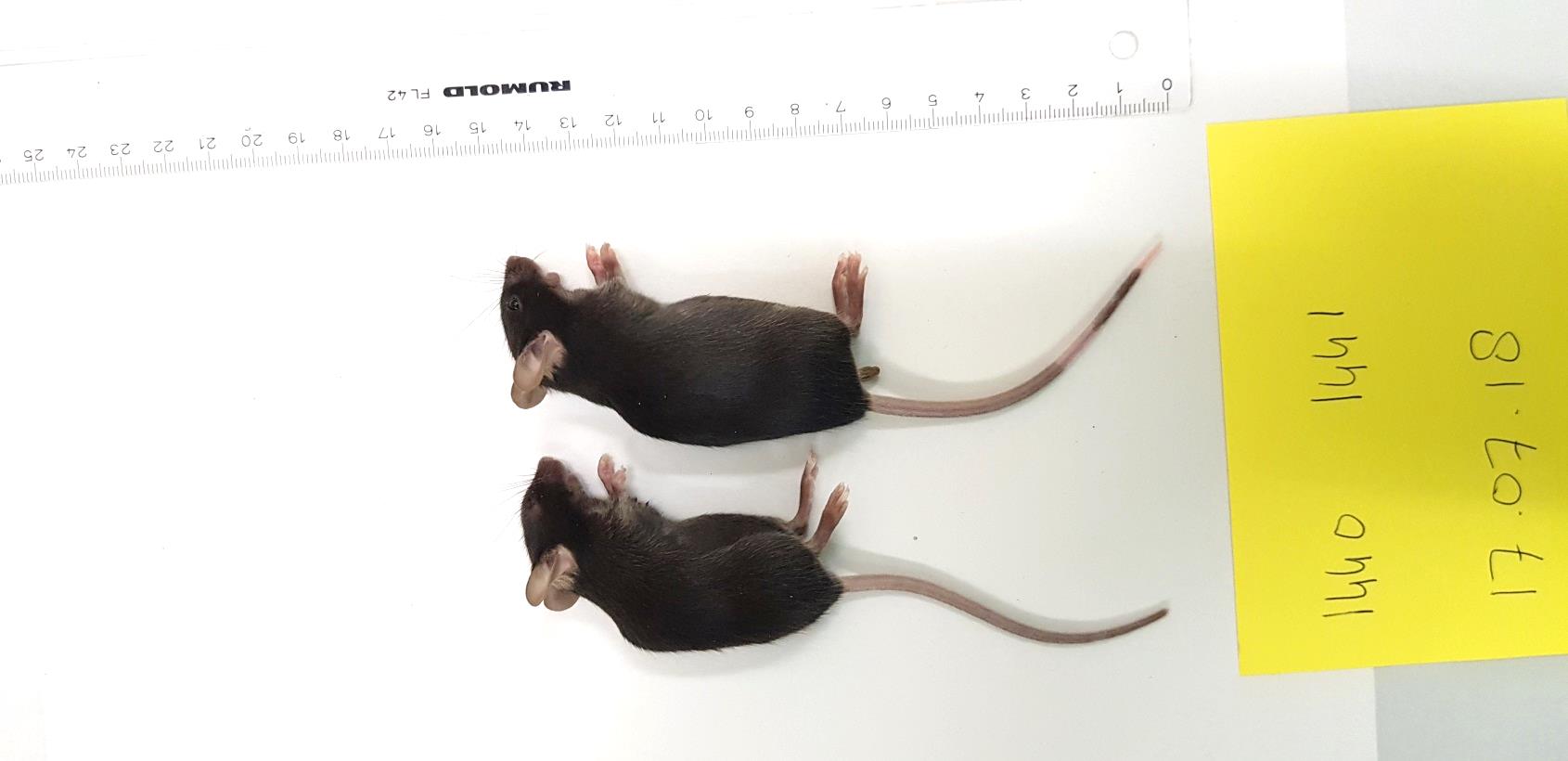

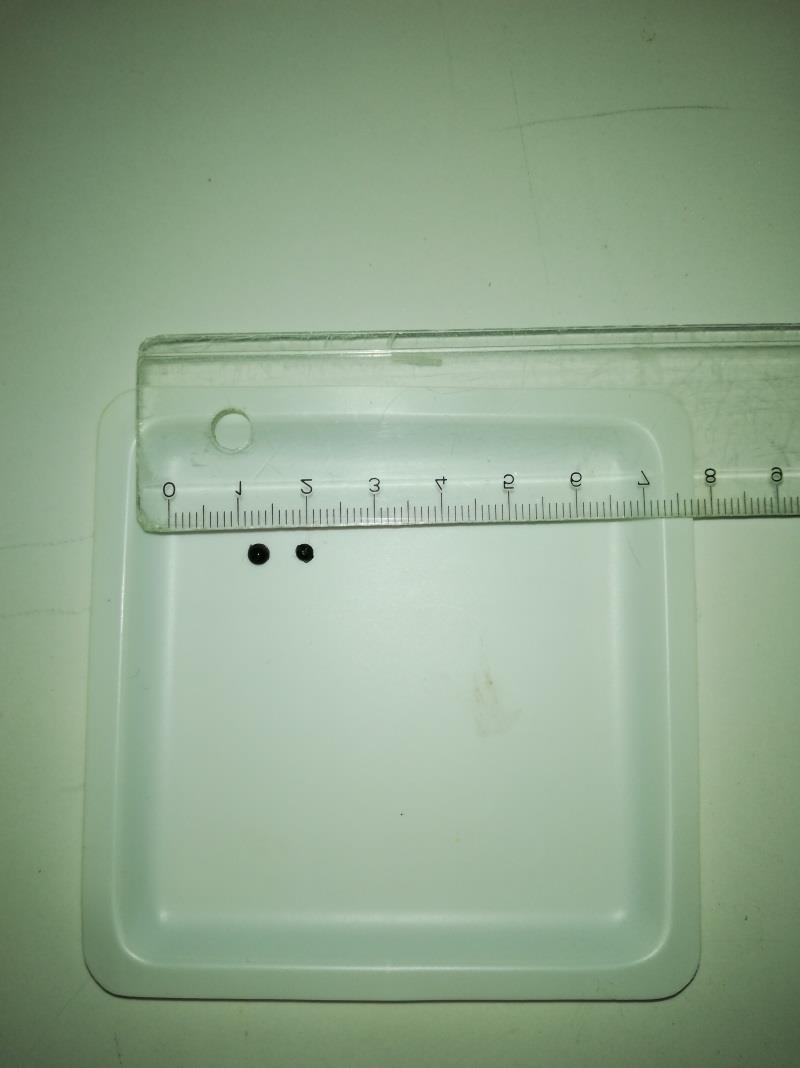


**D**


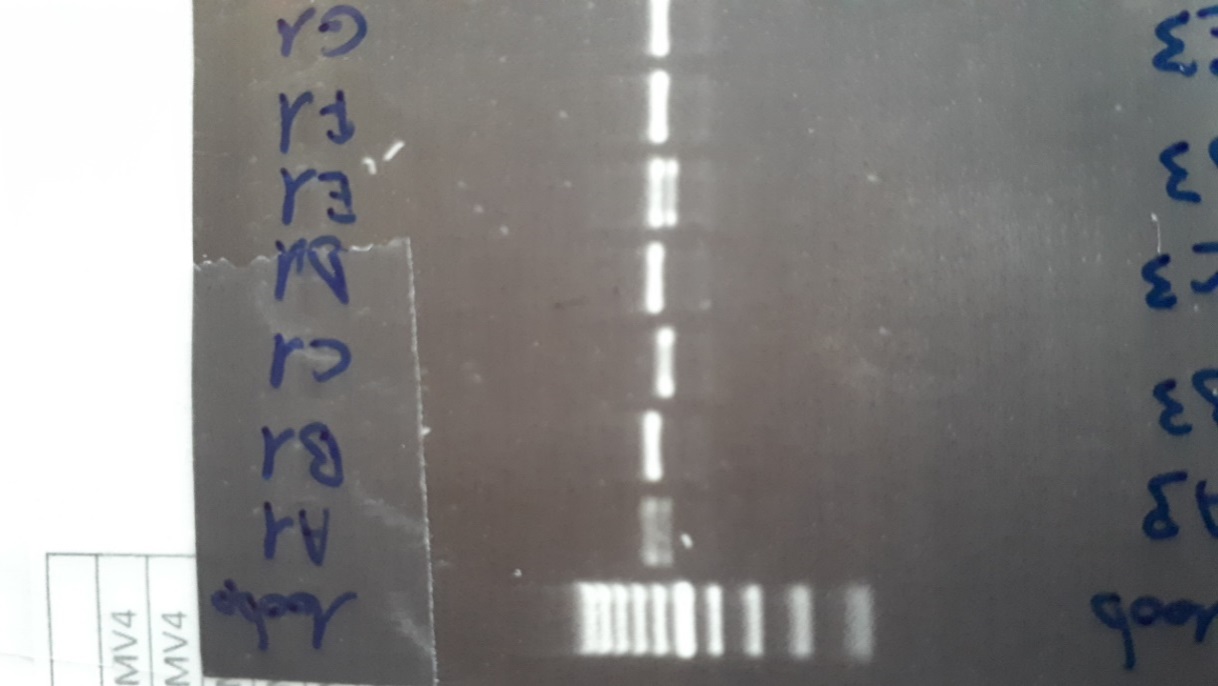


**XX^50ntdel^**

**XY**

**Parents:**

**4 female siblings**

**Supplemental data to FIGURE S5D**

**Full length gel**

**
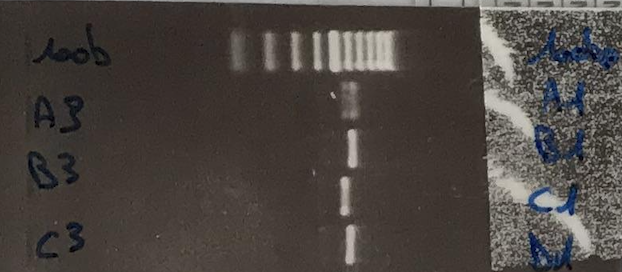
**

**FIGURE S6**

**
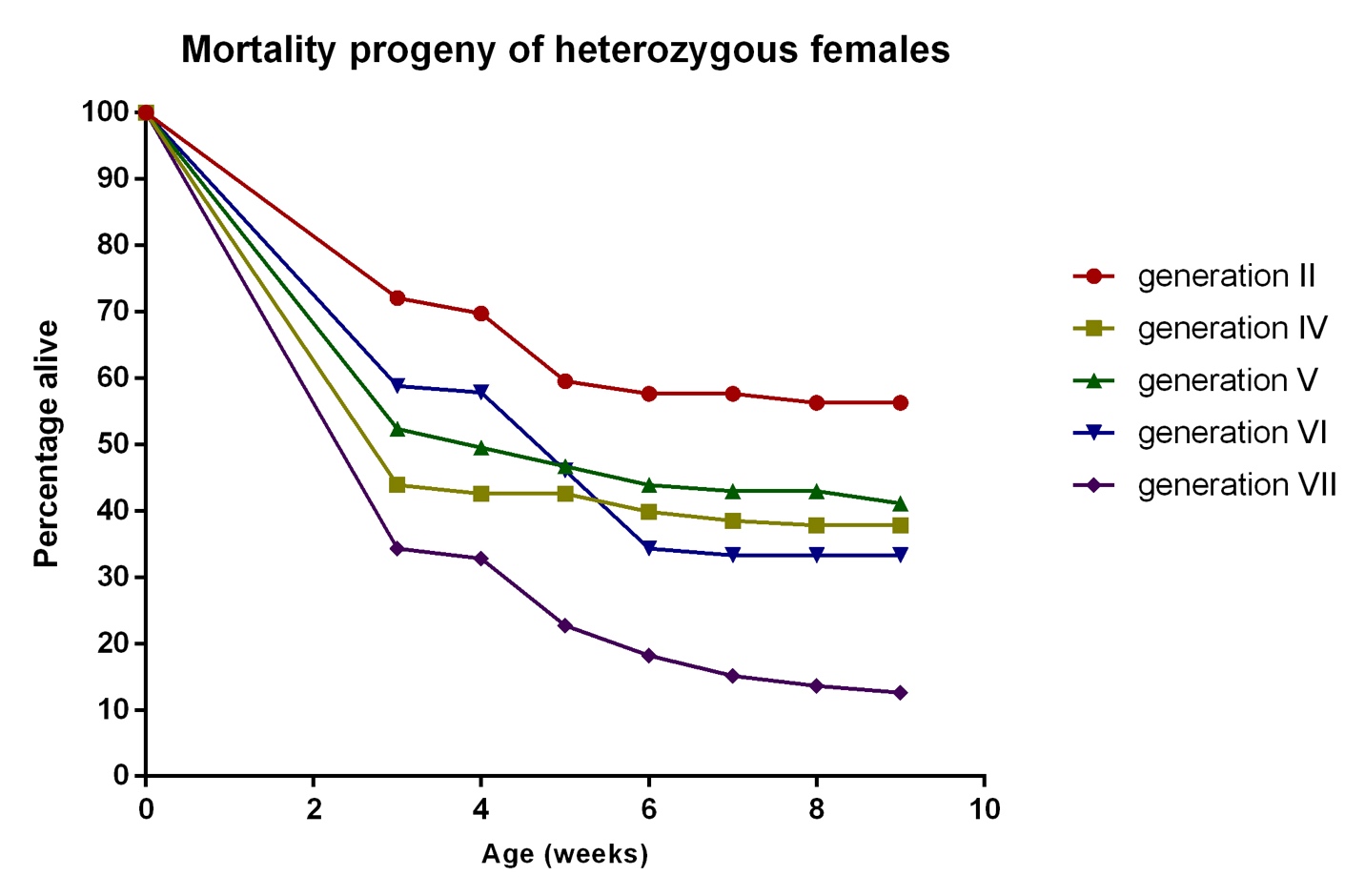

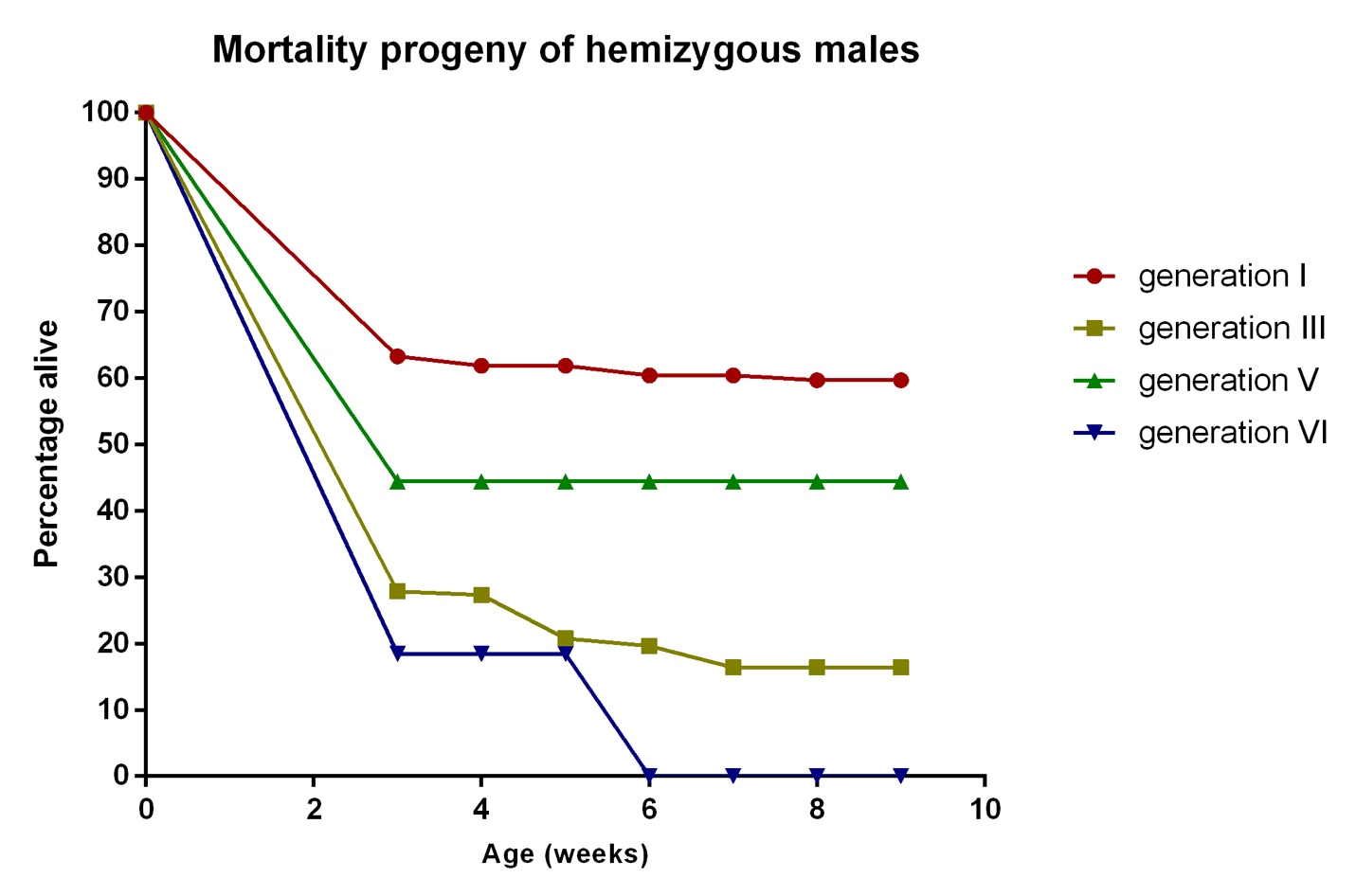
**

**B**

**A**

**Additional methods**

**Mice**

We quantified the beta cell (insulin positive cells) and pancreas area using the software Quantitative Pathology and Bioimage Analysis (QuPath, <https://qupath.github.io/>). We used two to three pancreatic sections at least 100 μm a part of three 3-week-old male mice (3 mutant and 3 non-mutant *Nexmif* mice) marked by immunofluorescent staining for insulin and DAPI.

**Zebrafish**

Experiments were carried out using the following zebrafish lines: Tubingen (WT), Tg(ptf1a:GFP;insa:mCherry) kindly received from Steve Leach (JHU/Mount Sinai), and Tg(insa:mCherry) and Tg(insa:nsfB-mCherry) kindly received from Michael Parsons (JHU/UCI).). Adult zebrafish were housed and naturally mated according to standard protocol. All zebrafish work was conducted in accordance with University of Maryland IACUC guidelines.

**Morpholinos**

Morpholinos (MOs) of targeted mRNAs were injected into one- to two-cell-stage embryos. We validated a splice-blocking MO to target the Nexmif transcript (e4i4 5′-TTAAGTGCTGAACTTACCGGGATTC-3′). A control nonspecific MO was used (5′-CCTCTTACCTCAGTTACAATTTATA-3′). The embryos were grown at 28 **°**C until harvesting for analyses. MO efficiency and lack of off-target toxicity were validated using qRT-PCR analysis for *nexmif* and p53 mRNA levels, respectively, compared with *actin* (primers available upon request).

**Beta-cell analysis**

The Tg(insa:mCherry) line which labels beta cells specifically by expressing mCherry under the control of the preproinsulin (insa) promoter was used to quantify the size of the beta-cell mass (area of mCherry expression) by imaging embryos using a Zeiss Lumar v12 stereomicroscope and ImageJ software as described [61]. The analysis of beta cells was performed on embryos collected from three different injections of either control or test MOs.

**Additional figure legends**

**Figure S1. Absence of Nexmif in mutant mice**

Generation of mice generated using CRISPR-Cas9 technology.

Nexmif localization (green) in mouse brain and pancreas in Nexmif non-mutant and mutant mice at postnatal day 0.

**Figure S2. Area of pancreatic beta-cells over total pancreas, body weight and beta-cell apoptosis**

1. Beta-cell area over total pancreas area is indicated in %, with a significant difference ( P= 0.0423) between *Nexmif* non-mutant (n = 3) and mutant (n = 3) mice at postnatal day 21. N = number of mice, in means  ± SD. Unpaired Student t-test.
2. Body weight is indicated in grams, in means  ± SD. Unpaired Student t-test. P = 0.38
3. Analysis of beta-cell apoptosis using the TUNEL assay. Results are expressed as the number of Tunel-positive cells as a percentage of insulin-positive cells, in means  ± SD. Unpaired Student t-test.

**Figure S3. Islet function after *nexmif* knockdown in zebrafish**

1. Beta-cell count in zebrafish in controls (4 ng control non-specific morpholino) and after 4 ng *nexmif*-specific morpholino treatment, 5 dpf.
2. Glucose-induced beta-cell expansion rate in zebrafish in controls (4 ng non-specific morpholino) and after 4 ng *nexmif*-specific morpholino treatment.

**Figure S4. Volcano plot showing RNA sequencing data**

A total of 3129 genes were differentially regulated (in red): 2418 genes were downregulated and 711 upregulated. The x-axis represents the fold changes (log_2_) and the y-axis -log_10_ of the *P* values.

**Figure S5. Observed phenotypes in the *Nexmif* mutant mouse colony**

1. 5-week-old *Nexmif* mutant male showing growth retardation, curved spine, and low-set ears.
2. 3-week-old *Nexmif* non-mutant male mouse with polydactyly of the left hind foot and growth retardation.
3. 8-week-old *Nexmif* heterozygous mutant female with micropthalmia. Inset: a small eye versus a normal eye.
4. PCR results of 4 female siblings indicating the total or partial lack of one X chromosome in one female and the copy numbers of three X-linked genes (*Nexmif*, *Atrx*, and *Bmx*) in that female in comparison with a control female and a control male.

**Figure S6. Mortality rates in the *Nexmif* mouse colony**

1. Surviving percentages of the progeny of male *Nexmif* mutant mice from generations 1, 3, 5, and 6.
2. Surviving percentages of the progeny of female heterozygous *Nexmif* mutant mice from generations 2, 4, 5, 6, and 7.

**Additional Table S1: Gene ontology analysis of RNA sequencing data**

**See attached Excel files**

**Additional Table S2: Primary and secondary antibodies**

| **Primary ANTIBODY** | **DILUTION** | **REFERENCE** | **Secondary ANTIBODY** | **Species** |
| --- | --- | --- | --- | --- |
| NEXMIF | 1:200 | Abcam ab150784 | Alexa Fluor® 488 | rabbit |
| KI67 | 1:300 | Invitrogen 41-5698-82 | eFluor® 570 | rat |
| INSULIN | 1:1000 | Sigma I2018 | Alexa Fluor® 647 | mouse |
| G3BP1 | 1:100 | Santa Cruz sc-365338 | Alexa Fluor® 594 | mouse |
| γH2AX | 1:200 | Abcam ab26350 | Alexa Fluor® 594 | mouse |
| HISTONE H3.3 | 1:200 | Abcam ab62642 | Alexa Fluor® 594 | rabbit |
| ORF1 | 1:200 | Wenda et al., Developmental Cell 2017 | Alexa Fluor® 488 | rabbit |

Wenda, J.M., Homolka, D., Yang, Z., Spinelli, P., Sachidanandam, R., Pandey, R.R., and Pillai, R.S. (2017). Distinct Roles of RNA Helicases MVH and TDRD9 in PIWI Slicing-Triggered Mammalian piRNA Biogenesis and Function. Developmental Cell *41*, 623-637.e9.

**Additional Table S3. qPCR Primers used in this study**
